# Serum Osteocalcin and CTX-I in Fit-to-Race Thoroughbred Racehorses: Reference Intervals and Associations with Demographic, Training, and Lameness-Related Factors

**DOI:** 10.3390/ani16162514

**Published:** 2026-08-12

**Authors:** Peter Tually, Jack Meadows, Matilda Hathway, Geoffrey Currie

**Affiliations:** 1 Department of Nuclear Medicine, TeleMedVET, 44 Epsom Ave, Ascot, WA 6104, Australia; j.meadows@telemed.net.au; 2 School of Dentistry and Medical Sciences, Charles Sturt University, Wagga Wagga, NSW 2678, Australia; gcurrie@csu.edu.au; 3 School of Veterinary Nursing, AVT, 39 Epsom Avenue, Ascot, WA 6104, Australia; matilda.hathway@gmail.com

**Keywords:** equine athlete, osteocalcin, C-terminal telopeptide, skeletal remodelling, lameness, musculoskeletal injury, injury risk, diagnostic screening, racehorse welfare, polytrack, thoroughbred

## Abstract

Musculoskeletal injury remains a major welfare and performance concern in Thoroughbred racing, and biomarkers for early risk stratification are of scientific interest. This multicentre observational cohort study characterised serum concentrations of two bone turnover markers, osteocalcin/BGLAP (OC) and C-terminal telopeptide of type I collagen (CTX-I), in 1359 fit-to-race Thoroughbred racehorses sampled across New South Wales, Victoria, and Western Australia. Biomarker distributions, population-wide percentile ranges, demographic and training-related associations, and short-term soundness outcomes were evaluated using nonparametric methods. Both markers were positively skewed, with population-wide percentile ranges (2.5–97.5 percentile) of 0.13–9.44 ng/mL for CTX-I and 0.02–7.25 ng/mL for OC; CTX-I varied substantially by jurisdiction, so this pooled range should not be interpreted as a clinical reference interval for individual horses. CTX-I varied significantly by age, jurisdiction, venue, and training surface, with markedly higher concentrations in New South Wales horses and lower concentrations associated with polytrack training, but did not predict lameness outcomes. OC concentrations were significantly higher in horses classified as lame at sampling and showed modest discriminatory ability for subsequent lameness in univariate analysis, with the strongest performance for persistent lameness at both 7 and 28 days; this association attenuated after adjustment for age, sex, state, and training surface. At an unadjusted operational threshold of approximately 1.24 ng/mL, OC achieved high negative predictive value for persistent lameness. These findings suggest OC may have scientific value as a potential rule-out screening marker for short-term soundness, warranting further investigation, while CTX-I appears more informative for characterising population-level skeletal turnover variation.

## Simple Summary

Racehorses can develop bone and limb problems during training before obvious signs of injury are seen. Detecting horses at lower or higher short-term risk could help trainers and veterinarians make safer decisions and improve horse welfare. This study examined whether two simple blood tests that reflect bone activity could help assess soundness risk in Thoroughbred racehorses in active training. Blood samples from 1359 fit-to-race horses in New South Wales, Victoria, and Western Australia were tested for osteocalcin, a marker linked with new bone formation, and C-terminal telopeptide of type I collagen, a marker linked with bone breakdown and repair. Osteocalcin was higher in horses that were lame at the time of sampling and was also associated with short-term lameness over the following 7 and 28 days. A low osteocalcin result was linked to a lower likelihood of persistent lameness, although this link weakened once other factors such as age, sex, and training conditions were taken into account. The second marker varied strongly between locations and training surfaces but did not predict lameness. These findings suggest osteocalcin is a promising marker for further scientific research into soundness risk in this population, although its clinical usefulness has not yet been established.

## 1. Introduction

Musculoskeletal injury remains one of the most important welfare, performance, and public-confidence challenges in Thoroughbred racing, contributing to training interruption, race-day fatalities, and associated jockey injury risk [1,2]. Racehorses are exposed to repeated high-speed loading during training and competition, and although this loading is essential for skeletal adaptation, it can also contribute to accumulated microdamage when the balance between damage, repair, and recovery is disrupted [3,4,5]. A major difficulty for injury prevention is that the biological changes preceding clinically apparent lameness or more severe injury may develop before overt signs are evident during routine clinical assessment [2]. Consequently, there is a need for practical, scalable tools that can complement veterinary examination and help identify horses at increased short-term risk while they are still in active training [6,7].

Bone is a dynamic tissue that responds continuously to mechanical load through coupled processes of formation and resorption [8,9]. In athletic horses, appropriate training stimulates skeletal modelling and remodelling, whereas excessive or poorly timed loading may exceed the capacity for repair [4]. Advanced imaging can detect bone stress and focal pathology, but its cost, logistical demands, and limited suitability for repeated population-level screening restrict routine use in large training cohorts [10,11]. Blood-based bone turnover markers may therefore provide a useful adjunct by offering a minimally invasive indication of underlying skeletal metabolic activity [12,13].

Osteocalcin (OC) is associated with osteoblastic activity and bone formation and is commonly interpreted as a marker of active bone formation and remodelling [14]. C-terminal telopeptide of type I collagen, or CTX, is released during collagen degradation and reflects bone resorption. Considered together, these markers provide complementary information about the balance between bone formation and bone resorption, a balance that is central to maintaining skeletal integrity in racehorses. However, interpretation of these markers in field populations is complicated by biological, demographic, environmental, and training-related influences. Age, sex, jurisdiction, training surface, time in work, and recent training intensity may all affect bone turnover, making it necessary to define population behaviour before biomarkers can be applied clinically [7,15].

Previous work has begun to validate this approach by comparing circulating bone turnover markers with objective imaging findings. In a prospective observational case-control study of 96 racehorses, including 48 lame horses referred for scintigraphy and 48 clinically sound controls, serum OC was higher in lame horses than in controls and differed across scintigraphic categories [16]. Horses with no abnormality detected on scintigraphy had lower OC concentrations than horses with positive scans, and exploratory pooled analysis showed that higher OC was associated with musculoskeletal injury status. In that study, CTX and the CTX:OC ratio were not significantly associated with short-term adverse musculoskeletal outcomes in clinically sound horses. A subsequent pooled two-phase cohort incorporating quantitative radiomic analysis of nuclear scintigraphy similarly found that OC differed significantly across scan categories, whereas CTX alone did not show the same pattern. This subsequent study also suggested that combining imaging-derived asymmetry indices with OC may improve risk stratification of exercise-induced bone pathology in racehorses [16].

Together, these previous studies support the biological plausibility of OC as a marker of active skeletal remodelling associated with clinically relevant bone pathology. However, both studies were centred on horses undergoing scintigraphic investigation or smaller controlled cohorts. Translation into routine racing practice requires evaluation in a much larger field population of actively training horses, including horses considered fit for training at the time of sampling. The present study was therefore designed to extend this earlier work from imaging-associated case-control and pooled scintigraphy cohorts into a large, multi-jurisdictional surveillance population.

The aim of this study was to characterise, at a population level, serum OC and CTX concentrations in a large cohort of fit-to-race Thoroughbred racehorses in active training across New South Wales, Victoria, and Western Australia. The primary objective was to establish population-level distributions and reference intervals for OC, CTX, and the CTX:OC ratio and to evaluate whether biomarker concentrations varied meaningfully according to demographic, geographic, and training-related variables. The secondary objective was to determine whether OC, CTX, or the CTX:OC ratio was associated with short-term operational outcomes, specifically whether horses remained sound and in active work at 7 and 28 days following sampling. We hypothesised that blood-based markers of bone turnover would vary across identifiable horse and training subgroups and that one or more markers would show a population-level association with short-term lameness risk.

## 2. Materials and Methods

This multicentre observational cohort study investigated circulating bone turnover markers in actively training Thoroughbred racehorses from three Australian racing jurisdictions. Blood samples were collected from clinically active racehorses under standardised field conditions and analysed centrally to determine serum concentrations of osteocalcin (OC; bone gamma-carboxyglutamate protein, BGLAP) and C-terminal telopeptide of type I collagen (CTX-I). Biomarker concentrations were compared with demographic characteristics, training variables, geographical location, and short-term clinical outcomes.

A total of 1359 Thoroughbred racehorses actively engaged in race training were recruited from metropolitan training centres in New South Wales, Victoria, and Western Australia as part of a collaborative Racing Australia bone health surveillance programme. Eligible horses were considered fit for training by their attending trainer at the time of blood collection. Horses represented a broad cross section of the Australian racing population, including 2-year-olds entering training through to older racehorses actively competing. Horses that were spelling or retired were excluded. The final study population comprised 493 horses from Western Australia, 481 from New South Wales, and 385 from Victoria.

Blood samples were obtained by jugular venepuncture under routine field conditions by experienced veterinary personnel or trained sampling staff. Each horse contributed a single blood sample to this dataset. Samples were collected into serum tubes, allowed to clot, centrifuged according to standard laboratory procedures, and stored frozen until analysis. Samples were transported to the central laboratory in insulated, ice-filled transport containers rather than in continuously temperature-monitored units, following the standardised protocol for equine biomarker studies. Upon arrival, samples were pro-cessed immediately, centrifuged, and serum stored at −80 °C until analysis. Because OC is a large, relatively stable peptide, an in-house validation study (*n* = 5 horses, University of Western Australia) assessed the effect of transport duration (1, 2, 3, 4, and 6 h) and collection tube type (clot-activator and plain tubes) on measured concentrations; no significant differences were observed, supporting the robustness of the transport protocol. Serum concentrations of OC and CTX-I were quantified using commercially available enzyme-linked immunosorbent assay kit according to the manufacturer’s instructions (C-terminal telopeptides of type I collagen, CTX; osteocalcin, OC; MyBioSource, San Diego, CA, USA). Laboratory personnel were blinded to horse identity and subsequent outcome status throughout sample analysis. Values below the analytical limit of detection were treated as missing observations.

For each horse, demographic and management information were recorded at the time of sampling. Variables included age, sex, state, training venue, main training surface, morning work type, morning training surface, weeks in work, days since last fast work, and ambient temperature at sampling. Where available, trainer-reported health records classified horses into one of six musculoskeletal categories: sound/bad mover, lame, historic/treating, shinsore, kissing spine/back, or unknown; as with the follow-up outcome data described below, these classifications were not independently confirmed by veterinary examination. Horses classified as kissing spine/back were considered a distinct category from lame, reflecting the clinical convention that horses with overriding dorsal spinous processes may continue racing successfully following medical management and are therefore not classified as lame at the time of sampling. Binary comparisons were restricted to horses classified as sound or lame.

Following blood collection, horses were monitored for short-term training continuity. Outcome status was recorded at both 7 and 28 days after sampling by the attending trainer using predefined categories: sound, lame, spelling, unknown, and sold/departed. Primary analyses compared horses remaining sound with horses classified as lame. Horses recorded as spelling, unknown, or sold/departed were excluded from binary outcome analyses. Persistent lameness was defined as classification as lame at both the 7-day and 28-day follow-up assessments. The overlap between 7-day and 28-day lameness classifications was examined descriptively using cross-tabulation. Subsequent racing activity was also recorded as the number of races between 28 and 56 days following sampling and total races within 60 days.

### 2.1. Statistical Methods

All statistical analyses were performed using IBM SPSS Statistics version 26 (IBM Corp., Armonk, NY, USA). Prior to analysis, data were examined for completeness and consistency. Horse identifier codes, geographic designations, and categorical variables were converted to numerical codes with value labels applied in SPSS Variable View. Values at or below the assay limit of detection were treated as system-missing. The final analytical dataset comprised *n* = 1359 horses; valid biomarker data were available for *n* = 1345 horses (CTX) and *n* = 1344 horses (OC/BGLAP).

Normality of biomarker distributions was assessed using the Kolmogorov–Smirnov test with Lilliefors correction and the Shapiro–Wilk test. Given confirmed non-normality for both markers (*p* < 0.001 for both), all subsequent group comparisons employed nonparametric methods. Differences across categorical subgroups (age group, sex, state, training venue, recent lameness status, main training surface, morning work type, and morning training surface) were assessed using the Kruskal–Wallis H test. Where significant, post hoc pairwise comparisons were performed using the Mann–Whitney U test with Bonferroni correction. Median concentrations and interquartile ranges are reported for each subgroup alongside the corresponding mean ranks; 95% confidence intervals for these medians were estimated using a percentile bootstrap (2000 resamples).

Associations between biomarker concentrations and continuous variables (weeks in work, days since last fast work, and ambient temperature) were assessed using Spearman’s rank-order correlation (r_s_). The relationship between CTX and OC/BGLAP was assessed in the same manner. An exploratory CTX:OC ratio was computed as CTX divided by OC for horses with valid values for both markers (*n* = 1330), as an index of the resorption-to-formation balance. The ratio was evaluated descriptively, in relation to geographic variation, concurrent lameness status, and post-sampling soundness outcomes, recognising its highly skewed distribution and exploratory status.

Population reference intervals (RI) for CTX and OC/BGLAP were established nonparametrically as the central 95% of the distribution, defined by the 2.5 and 97.5 percentiles calculated using the weighted average method (HAVERAGE) in SPSS v26, consistent with ASVCP reference interval guidelines [17]. This was a descriptive, reference-interval-generating study rather than a hypothesis-driven comparison with a single pre-specified primary endpoint; consistent with ASVCP RI guidelines, a 95% interval was used as the standard convention for veterinary RIs, and sample size (*n* = 1359) reflected the available operational dataset rather than an a priori power calculation. The 95% confidence intervals for the lower and upper RI limits were calculated using the rank-based Reed method [18]. Given that the reference population included a small proportion of horses classified as lame at the time of sampling, a sensitivity analysis was performed in which RIs were recalculated, restricted to horses classified as sound at sampling, to assess whether their inclusion materially affected the reported ranges. RIs were also stratified by state (Western Australia, New South Wales, and Victoria), given the substantial geographic variation in biomarker concentrations identified in subgroup analyses (Section 3.4). For these sensitivity and stratified analyses, 95% confidence intervals for the RIs were estimated using a percentile bootstrap (2000 resamples) rather than the Reed method used for the primary pooled RI.

Associations between biomarker concentrations at sampling and subsequent soundness outcomes were evaluated using the Mann–Whitney U test, with analyses restricted to horses classified as sound (code 1) or lame (code 2) at 7 and 28 days post-sampling; horses recorded as spelling, unknown, or sold/departed were excluded. The discriminatory ability of CTX and OC/BGLAP for predicting lameness was assessed using receiver operating characteristic (ROC) curve analysis; the area under the curve (AUC) was reported with asymptotic 95% confidence intervals under the nonparametric assumption (AUC = 0.5 indicating no discrimination; AUC = 1.0 indicating perfect discrimination). Four outcome definitions were evaluated: (a) 7-day lameness only; (b) 7-day lameness or spelling; (c) 28-day lameness or spelling; and (d) persistent lameness, defined as lame at both 7-day and 28-day follow-up. The optimal OC cutoff for each outcome was identified by maximising the Youden index (sensitivity + specificity − 1) from the ROC coordinate tables. At the optimal cutoff, sensitivity, specificity, positive predictive value (PPV), and negative predictive value (NPV) were derived from 2 × 2 contingency tables; 95% confidence intervals for all four proportions were calculated using the Wilson score method. To assess whether the univariate association between OC and 7-day lameness status was independent of other subgroup variables, a multivariable binary logistic regression model was constructed with 7-day outcome (sound vs. lame) as the dependent variable and OC concentration, age group, sex, state, and main training surface as predictors. Sex categories with very small sample sizes (rig, *n* = 2; stallion, *n* = 4 within the analytic sample) were excluded prior to modelling, as their inclusion resulted in quasi-complete separation and model nonconvergence; the final model was restricted to horses classified as filly, mare, colt, or gelding. Two-way interaction terms (age group × sex; recent lameness status × main training surface) were considered but not included in the final model, given the substantial reduction in subgroup sample sizes and consequent risk of estimation instability. Adjusted odds ratios with 95% confidence intervals are reported.

Associations between biomarker concentrations and subsequent racing activity (races between 28 and 56 days and races within 60 days post-sampling) were assessed using Spearman’s rank-order correlation. All tests were two-tailed. Statistical significance was set at *p* < 0.05 unless otherwise specified by Bonferroni correction.

All animal procedures performed in this study were reviewed and approved by the TeleMedVET Animal Ethics Committee (Approval No. TMV-0006). The study was conducted in accordance with the Committee’s animal welfare requirements and ethical standards for the use of animals in research.

### 2.2. Declaration of Generative AI and AI-Assisted Technologies

During preparation of this manuscript, the authors used Julius AI to assist with editorial refinement, reference checking, and identification of duplicate or redundant records. The tool was not used to generate original data, determine study eligibility, perform statistical analysis, replace author judgment, or independently interpret findings. All AI-assisted outputs were reviewed, verified, edited, and approved by the authors, who take full responsibility for the final manuscript.

## 3. Results

### 3.1. Sample Characteristics

Blood samples were collected from 1359 fit-to-race Thoroughbred racehorses across three Australian states; state distribution, sex, and age are summarised in Table 1. Mean age was 3.52 years (SD = 1.40). Valid CTX values were available for 1345 horses, and valid OC values were available for 1344 horses. Training characteristics (weeks in work, days since last fast work, and ambient temperature at sampling) are summarized in Table 1. The remaining 531 horses provided only demographic and biomarker data, reflecting variation in data collection completeness across sites. Extended values for weeks in work and days since last fast work were confirmed by field workers as consistent with horses returning from spelling, in accordance with the study definition of fit-to-work rather than fit-to-race. Lameness status at sampling and follow-up outcomes at 7 and 28 days are summarised in Table 1. All follow-up data were self-reported by trainers and could not be independently verified by veterinary examination.

### 3.2. Biomarker Distribution

Serum CTX concentrations were available for 1345 horses (missing *n* = 14) and ranged from 0.01 to 24.66 ng/mL (mean = 2.47 ng/mL, SD = 2.85; median = 1.15 ng/mL, IQR = 3.40). The distribution was markedly positively skewed (skewness = 2.51, SE = 0.067) with substantial excess kurtosis (kurtosis = 10.18, SE = 0.134) indicating a heavy right tail driven by a small number of horses with high resorption values. Serum OC concentrations were available for 1344 horses (missing *n* = 15) and ranged from 0.00 to 33.10 ng/mL (mean = 1.41 ng/mL, SD = 2.49; median = 0.75 ng/mL, IQR = 1.01). OC showed more extreme positive skew (skewness = 5.93, SE = 0.067) and leptokurtosis (kurtosis = 46.83, SE = 0.134), reflecting a concentration of values at the lower end of the scale with an extended upper tail. Formal testing confirmed significant departure from normality for both markers (Kolmogorov–Smirnov with Lilliefors correction: CTX D = 0.218, *p* < 0.001; OC D = 0.286, *p* < 0.001; Shapiro–Wilk: CTX W = 0.739, *p* < 0.001; OC W = 0.445, *p* < 0.001), supporting the use of nonparametric statistical methods throughout all subsequent analyses.

### 3.3. Reference Intervals

Reference intervals (RIs) were established using the nonparametric method, defined as the central 95% of the distribution (2.5 to 97.5 percentiles) following ASVCP/Reed et al. guidelines [17,18], with 95% confidence intervals calculated using the rank-based formula described in the Methods.

The RI for serum CTX was 0.13 to 9.44 ng/mL (95% CI for lower limit: 0.10–0.15 ng/mL; 95% CI for upper limit: 8.10–10.18 ng/mL). The RI for serum OC was 0.02 to 7.25 ng/mL (95% CI for lower limit: 0.01–0.05 ng/mL; 95% CI for upper limit: 5.68–10.66 ng/mL). The wide confidence interval for the OC upper reference limit reflects elevated variability in the upper tail of the distribution, driven by a small number of horses with markedly elevated OC concentrations. Both reference intervals were wide, reflecting genuine biological variability across a large, heterogeneous population spanning multiple states, training environments, age groups, and sexes. Given the significant geographic variation in biomarker concentrations identified in subgroup analyses (Section 3.4.3), population-wide reference intervals should be interpreted with caution when applied to individual state or regional populations. The resulting reference intervals are presented in Table 2.

To assess whether inclusion of horses classified as lame at sampling materially affected the reported RIs, a sensitivity analysis restricted to horses classified as sound at sampling was performed (CTX *n* = 694; OC *n* = 698). The resulting RIs were closely comparable to those derived from the full cohort: CTX 0.12 (95% CI 0.09–0.15) to 9.76 (95% CI 8.08–11.10) ng/mL and OC 0.015 (95% CI 0.010–0.034) to 7.12 (95% CI 4.46–11.57) ng/mL. All sound-only RIs fell within the 95% confidence intervals of the corresponding full-cohort limits, indicating that inclusion of a small proportion of lame horses did not materially affect the reported RIs.

Given the substantial geographic variation identified in subgroup analyses (Section 3.4.3), RIs were also calculated separately by state. For CTX, the 2.5–97.5 percentile range was 0.14 (95% CI 0.06–0.18) to 8.38 (95% CI 7.65–9.98) ng/mL in Western Australia, 0.12 (95% CI 0.09–0.18) to 11.92 (95% CI 9.43–17.44) ng/mL in New South Wales, and 0.11 (95% CI 0.09–0.17) to 7.27 (95% CI 6.34–8.44) ng/mL in Victoria. For OC, the corresponding ranges were 0.008 (95% CI 0.005–0.010) to 9.34 (95% CI 4.65–11.57) ng/mL in Western Australia, 0.117 (95% CI 0.070–0.147) to 8.67 (95% CI 5.02–13.28) ng/mL in New South Wales, and 0.104 (95% CI 0.086–0.136) to 6.06 (95% CI 4.98–7.96) ng/mL in Victoria. Unlike the sound-only sensitivity analysis, state-stratified RIs showed meaningful divergence, particularly for the CTX upper limit (non-overlapping between New South Wales and Victoria) and the OC lower limit in Western Australia, which was an order of magnitude lower than either other state, supporting the use of jurisdiction-specific interpretive ranges rather than a single pooled interval.

### 3.4. Subgroup Comparisons

#### 3.4.1. Age Group

CTX concentrations differed significantly across age groups (Kruskal–Wallis H = 9.751, df = 3, *p* = 0.021). Post hoc pairwise comparisons using Mann–Whitney U tests with Bonferroni correction (adjusted α = 0.0083) identified a single significant difference: CTX was higher in young horses (1–2 years; median = 1.36 ng/mL, *n* = 343) compared with mid-age horses (3 years; median = 0.97 ng/mL, *n* = 439; U = 65,767, Z = −3.038, *p* = 0.002). No other pairwise comparisons reached the Bonferroni-corrected threshold. OC concentrations did not differ significantly across age groups (Kruskal–Wallis H = 1.764, df = 3, *p* = 0.623), and no post hoc comparisons were performed.

#### 3.4.2. Sex

CTX concentrations did not differ significantly across sex categories (Kruskal–Wallis H = 7.478, df = 5, *p* = 0.187), and no post hoc comparisons were performed. OC concentrations differed significantly across sex categories (Kruskal–Wallis H = 11.991, df = 5, *p* = 0.035). Post hoc pairwise Mann–Whitney U tests with Bonferroni correction (adjusted α = 0.005) identified a single significant difference: OC was higher in colts (median = 0.93 ng/mL, *n* = 158) compared with fillies (median = 0.69 ng/mL, *n* = 371; U = 24,171, Z = −3.193, *p* = 0.001). No other pairwise comparisons reached the Bonferroni-corrected threshold. Results for rigs (*n* = 2) are reported descriptively only and were excluded from post hoc comparisons due to insufficient sample size.

#### 3.4.3. Geographic Variation

Bone turnover marker (BTM) concentrations varied significantly by state for CTX (Kruskal–Wallis H = 155.656, df = 2, *p* < 0.001) and OC (H = 6.816, df = 2, *p* = 0.033). Post hoc Mann–Whitney U tests with Bonferroni correction (adjusted α = 0.0167) revealed that CTX differed significantly between all three state pairs: NSW horses had markedly higher CTX concentrations (median = 3.21 ng/mL) than both WA (median = 0.65 ng/mL; U = 65,255.5, Z = −11.626, *p* < 0.001) and VIC horses (median = 1.07 ng/mL; U = 62,739.0, Z = −7.963, *p* < 0.001), with WA also lower than VIC (U = 72,692.0, Z = −5.571, *p* < 0.001). For OC, no pairwise state comparisons survived the Bonferroni correction (all *p* > 0.017), suggesting that the overall significant Kruskal–Wallis result reflects marginal rather than robust state-level differences in bone formation activity.

At the venue level, CTX and OC concentrations differed significantly across the 12 collection sites (CTX: H = 524.976, df = 11, *p* < 0.001; OC: H = 131.422, df = 11, *p* < 0.001). Within NSW, Newcastle (CTX median = 4.80 ng/mL), Warwick Farm (3.40 ng/mL), and Bong Bong (2.23 ng/mL) showed markedly higher CTX than Rosehill (0.54 ng/mL), which was more consistent with WA and VIC venues. Within VIC, Cranbourne showed higher CTX (median = 1.95 ng/mL) than Flemington (0.99 ng/mL) and Ballarat (0.59 ng/mL). Albany (WA, *n* = 1) is reported descriptively only and excluded from interpretation. The geographic variation in CTX, particularly the divergence between NSW venues, warrants consideration when applying reference intervals across states.

#### 3.4.4. Recent Lameness at Time of Sampling

At the time of blood sampling, lameness status was available for 864 horses with valid CTX values and 867 with valid OC values. CTX concentrations did not differ significantly between horses classified as sound/bad mover (median = 0.94 ng/mL, *n* = 694) and those classified as lame (median = 1.20 ng/mL, *n* = 55; Mann–Whitney U = 16,164.5, Z = −1.891, *p* = 0.059). In contrast, OC concentrations were significantly higher in lame horses (median = 1.26 ng/mL, *n* = 57) compared with sound horses (median = 0.67 ng/mL, *n* = 698; U = 13,589.0, Z = −3.982, *p* < 0.001). This finding indicates that elevated OC at the time of sampling is associated with concurrent musculoskeletal lameness, suggesting active bone-forming responses to ongoing pathology. In contrast, CTX does not discriminate between soundness categories at the time of collection. Horses with unknown lameness status, historic or treated lameness, shinsore, or kissing spine/back pathology are reported descriptively only and were excluded from the binary comparison.

#### 3.4.5. Training Surface

CTX concentrations differed significantly by morning training surface (Kruskal–Wallis H = 32.162, df = 5, *p* < 0.001). Post hoc Mann–Whitney U tests with Bonferroni correction (adjusted α = 0.0033) identified polytrack as consistently associated with lower CTX compared with sand (U = 40,103.5, Z = −4.259, *p* < 0.001), grass (U = 3554.0, Z = −5.009, *p* < 0.001), and treadmill (U = 3407.0, Z = −3.117, *p* = 0.002). Additionally, CTX was higher in horses training on grass than water in the morning (U = 1008.0, Z = −3.104, *p* = 0.002). OC did not differ significantly by morning training surface (H = 4.689, df = 5, *p* = 0.455).

CTX also differed significantly by main training surface (Kruskal–Wallis H = 30.893, df = 2, *p* < 0.001). Post hoc comparisons with Bonferroni correction (adjusted α = 0.0167) showed that horses trained primarily on grass had significantly higher CTX than those on sand (median = 1.95 vs. 0.87 ng/mL; U = 11,072.5, Z = −4.919, *p* < 0.001) and poly (median = 1.95 vs. 0.86 ng/mL; U = 2553.0, Z = −5.588, *p* < 0.001). Sand and poly did not differ significantly (*p* = 0.054). OC did not differ significantly by main training surface (H = 1.481, df = 2, *p* = 0.477).

#### 3.4.6. Continuous Training Variables

Spearman’s rank correlations were used to examine associations between (BTM) concentrations and continuous training variables. CTX showed a small but statistically significant positive correlation with weeks in work (r_s_ = 0.080, *n* = 1019, *p* = 0.010), while correlations with days since last fast work (r_s_ = 0.064, *n* = 819, *p* = 0.066) and ambient temperature (r_s_ = 0.030, *n* = 1345, *p* = 0.269) were non-significant. OC showed a small but statistically significant positive correlation with ambient temperature (r_s_ = 0.081, *n* = 1344, *p* = 0.003), while correlations with weeks in work (r_s_ = −0.054, *n* = 1020, *p* = 0.087) and days since last fast work (r_s_ = 0.022, *n* = 821, *p* = 0.526) were non-significant. Despite reaching statistical significance in a large sample, the effect sizes for both CTX–weeks in work and OC–temperature associations were negligible (r_s_ < 0.10), indicating no practically meaningful relationships between these training variables and bone turnover markers (BTM) concentrations in this cohort. Full subgroup comparisons are presented in Table 3.

#### 3.4.7. Relationship Between CTX and OC/BGLAP

Serum CTX and OC concentrations showed a weak but statistically significant positive correlation across the full cohort (Spearman r_s_ = 0.157, *n* = 1330, *p* < 0.001), indicating that the two markers are largely independent and reflect different aspects of bone turnover. The low shared variance (r_s_^2^ ≈ 2.5%) confirms that CTX and OC provide complementary rather than redundant information in this population.

As an exploratory measure of the resorption-to-formation balance, a CTX:OC ratio was computed for horses with valid values for both markers (*n* = 1330; median = 2.04, range 0.01–1296.03). The distribution was extremely right-skewed (skewness = 21.2, kurtosis = 495.1), driven by a small number of horses with very low OC values, which produced extreme ratio values; the median is the appropriate measure of central tendency. As expected mathematically, the ratio correlated moderately with CTX (r_s_ = 0.625, *p* < 0.001) and inversely with OC (r_s_ = −0.608, *p* < 0.001), confirming it reflects both components.

The CTX:OC ratio differed significantly by state (Kruskal–Wallis H = 45.496, df = 2, *p* < 0.001). Notably, NSW horses showed the highest absolute CTX concentrations (Section 3.4.3) but an intermediate median ratio (3.26), suggesting proportional elevations of both resorption and formation markers in that state. WA horses showed the lowest ratio median (1.36) despite intermediate CTX values, indicating relatively greater formation activity; VIC horses showed the most balanced resorption-to-formation ratio (median = 1.48). These geographic differences in the ratio suggest that the bone remodelling balance, rather than resorption activity alone, varies across Australian racing jurisdictions.

**Figure 1:**
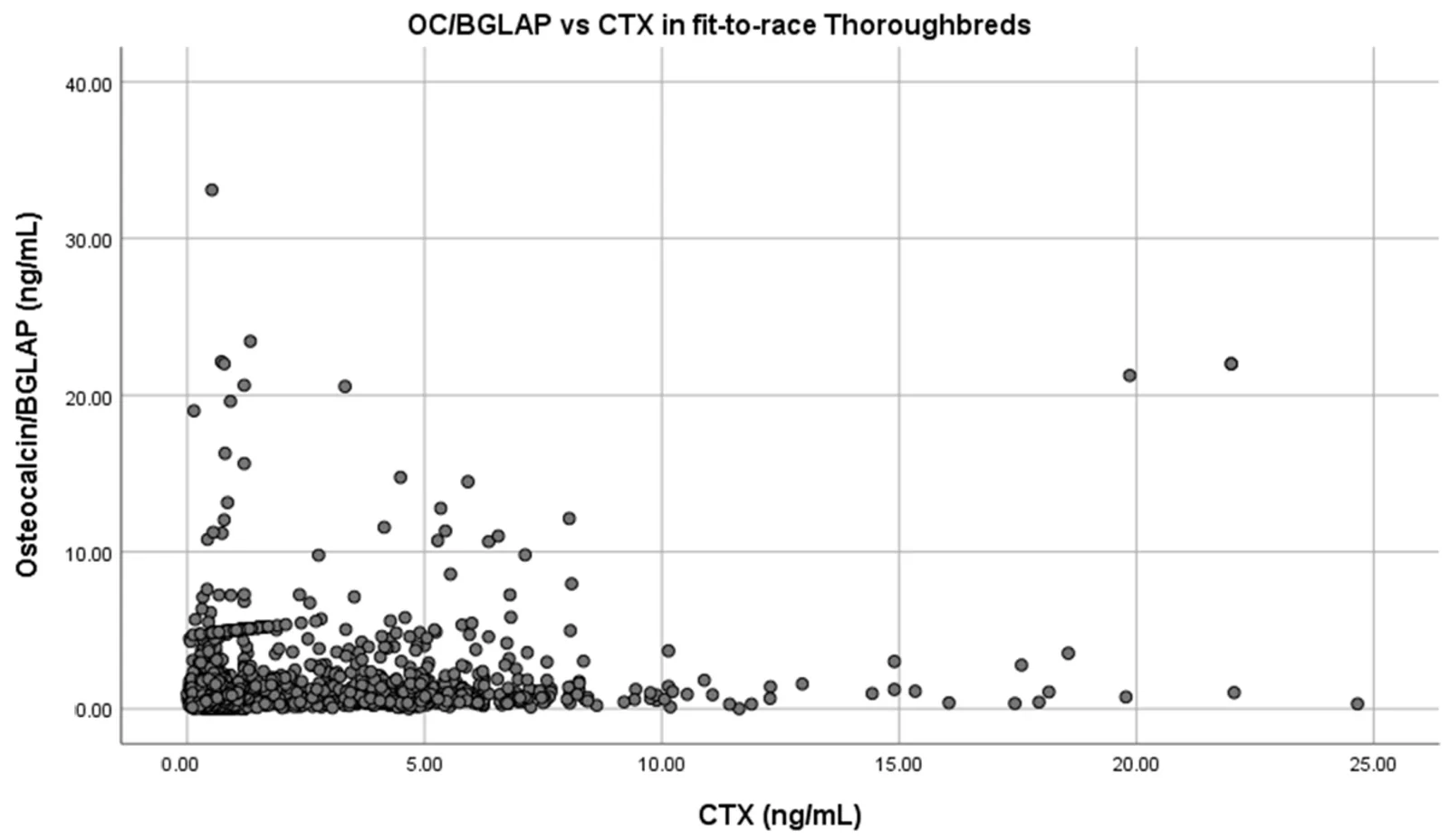
Scatterplot of OC/BGLAP vs. CTX concentrations in fit-to-race Thoroughbred racehorses (*n* = 1330).

The CTX:OC ratio did not differ significantly between horses classified as sound or poor movers (median = 1.94, *n* = 687) and those classified as lame at the time of sampling (median = 1.19, *n* = 55; U = 16,734.5, Z = −1.411, *p* = 0.158), indicating no discriminatory value of the ratio for concurrent lameness beyond OC alone. However, horses that were subsequently classified as lame at 7 days post-sampling showed a significantly lower ratio at the time of blood collection (median = 1.19, *n* = 121) compared with horses that remained sound (median = 2.09, *n* = 681; U = 34,352.0, Z = −2.917, *p* = 0.004), consistent with relatively elevated OC driving the ratio downward in horses at risk of short-term lameness. The relationship between OC/BGLAP and CTX concentrations is illustrated in Figure 1.

### 3.5. Outcome Analysis

#### 3.5.1. Post-Sample Soundness Outcomes

CTX concentrations did not differ significantly between horses that were sound (median = 0.89 ng/mL) and lame (median = 0.82 ng/mL) at 7 days post-sampling (Mann–Whitney U = 41,590.5, Z = −0.251, *p* = 0.802), nor between sound (median = 0.84 ng/mL) and lame horses (median = 0.84 ng/mL) at 28 days (U = 35,390.5, Z = −0.224, *p* = 0.823). In contrast, OC concentrations were significantly higher in horses classified as lame at 7 days (median = 0.99 ng/mL, *n* = 121) compared with sound horses (median = 0.62 ng/mL, *n* = 692; U = 30,956.5, Z = −4.578, *p* < 0.001) and in horses lame at 28 days (median = 0.84 ng/mL, *n* = 126) compared with sound horses (median = 0.66 ng/mL, *n* = 575; U = 29,785.5, Z = −3.128, *p* = 0.002).

ROC analysis confirmed that CTX had no discriminatory ability for either outcome (AUC = 0.499, 95% CI: 0.444–0.553, *p* = 0.961 for 7-day; AUC = 0.489, 95% CI: 0.434–0.545, *p* = 0.703 for 28-day). OC demonstrated modest but statistically significant discriminatory ability across all four outcome definitions. For 7-day lameness only, OC AUC = 0.619 (95% CI: 0.562–0.675, *p* < 0.001); for 7-day lameness or spelling, AUC = 0.644 (95% CI: 0.594–0.694, *p* < 0.001); for 28-day lameness or spelling, AUC = 0.568 (95% CI: 0.512–0.625, *p* = 0.014); and for persistent lameness at both 7 and 28 days, AUC = 0.687 (95% CI: 0.617–0.758, *p* < 0.001). The progressive increase in AUC from single timepoint to persistent lameness outcomes suggests that OC has greater discriminatory value for horses that remain lame over an extended period.

**Figure 2:**
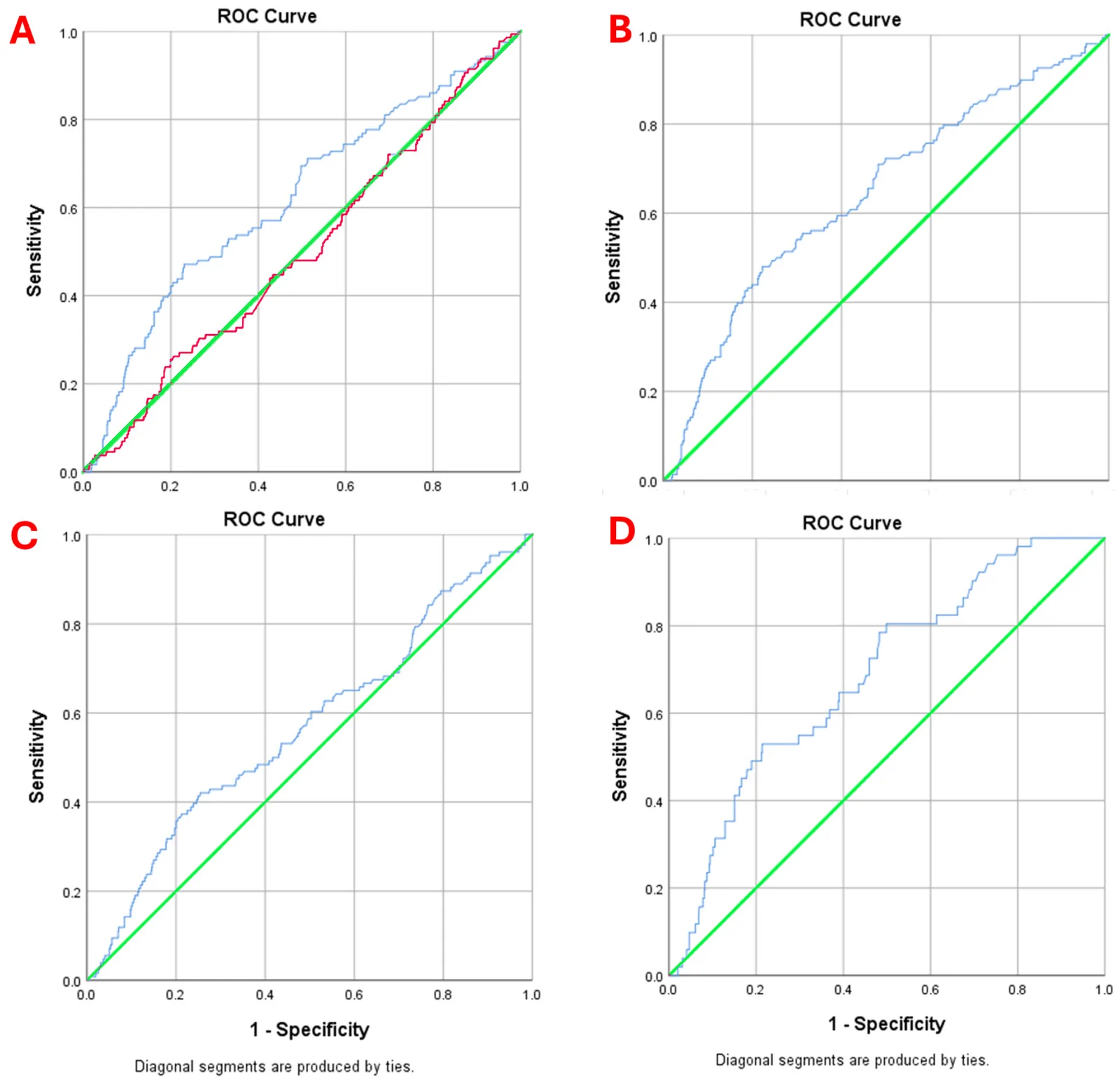
Receiver operating characteristic (ROC) curves for OC/BGLAP (blue) and CTX (red) predicting post-sample lameness outcome. (**A**) 7-day lameness only: OC/BGLAP AUC = 0.619 (95% CI 0.562–0.675, *p* < 0.001); CTX AUC = 0.499 (95% CI 0.444–0.553, *p* = 0.961). (**B**) 7-day lameness or spelling: OC/BGLAP AUC = 0.644 (95% CI 0.594–0.694, *p* < 0.001). (**C**) 28-day lameness or spelling: OC/BGLAP AUC = 0.568 (95% CI 0.512–0.625, *p* = 0.014). (**D**) Persistent lameness: OC/BGLAP AUC = 0.687 (95% CI 0.617–0.758, *p* < 0.001). Green diagonal represents chance discrimination (AUC = 0.5).

The Youden index identified consistent optimal OC cutoff values across all outcome definitions, clustering between 1.21 and 1.25 ng/mL. At a rounded operational threshold of approximately 1.24 ng/mL, OC demonstrated low-to-moderate sensitivity (0.421–0.529) and moderate specificity (0.744–0.786) across analyses. PPV was low across all analyses (0.154–0.316), reflecting the relatively low prevalence of lameness outcomes in this population. NPV was the strongest performance metric: 0.893 (95% CI: 0.865–0.915) for 7-day lameness, 0.875 (95% CI: 0.846–0.899) for 7-day lameness or spelling, 0.854 (95% CI: 0.821–0.882) for 28-day lameness or spelling, and 0.958 (95% CI: 0.938–0.971) for persistent lameness. These results indicate that OC below approximately 1.24 ng/mL at the time of sampling has reasonable utility as a rule-out marker for subsequent lameness, particularly persistent lameness, in fit-to-race Thoroughbreds. However, the modest AUC values and low PPV indicate that OC is not suitable as a standalone screening tool, and elevated OC should prompt further clinical or imaging investigation rather than serve as a definitive indicator of lameness risk. The discriminatory performance of OC/BGLAP and CTX for these outcomes is shown as ROC curves in Figure 2.

#### 3.5.2. Multivariable Analysis of OC and 7-Day Lameness

To assess whether the univariate association between OC and 7-day lameness status was independent of other subgroup variables, a multivariable binary logistic regression model was fitted (*n* = 805). After adjustment for age group, sex, state, and main training surface, OC concentration was no longer significantly associated with 7-day lameness (adjusted OR 1.042, 95% CI 0.979–1.110, *p* = 0.194), indicating that these covariates partly explained the univariate association. Age group (Mature vs. Young: OR 0.504, 95% CI 0.276–0.918, *p* = 0.025), sex (Mare vs. Filly: OR 1.839, 95% CI 1.042–3.245, *p* = 0.036), and main training surface (Poly vs. Sand: OR 0.343, 95% CI 0.123–0.960, *p* = 0.042) remained independently associated with 7-day lameness in the adjusted model.

#### 3.5.3. Association with Subsequent Racing Activity

Neither CTX nor OC at the time of blood sampling was significantly associated with subsequent racing activity. CTX showed no significant correlation with races between 28 and 56 days (Spearman r_s_ = 0.061, *n* = 836, *p* = 0.076) or races within 60 days (r_s_ = 0.060, *n* = 836, *p* = 0.085). OC similarly showed no significant correlation with races between 28 and 56 days (r_s_ = 0.039, *n* = 839, *p* = 0.254) or races within 60 days (r_s_ = 0.016, *n* = 839, *p* = 0.652). Effect sizes were negligible for all associations (r_s_ < 0.10), indicating that BTM concentrations at sampling are not meaningfully related to subsequent racing frequency in this cohort. These associations are summarized in Table 4.

## 4. Discussion

This study characterised serum concentrations of CTX and OC in a large, multi-jurisdictional cohort of Thoroughbred racehorses in active training. OC concentrations were higher in horses classified as lame at the time of sampling and showed modest but statistically significant discriminatory ability for subsequent lameness at 7 and 28 days. In contrast, CTX did not discriminate between sound and lame horses and had no useful predictive value for the short-term lameness outcomes assessed. CTX appears more strongly influenced by population, geographic, and training-surface effects than by short-term clinical outcome.

The elevation of OC in lame horses at the time of sampling is consistent with the interpretation that OC reflects active skeletal remodelling rather than structural bone strength or a specific diagnosis. Horses classified as lame had a median OC concentration of 1.26 ng/mL compared with 0.67 ng/mL in horses classified as sound or poor movers. OC also demonstrated significant, although modest, ROC performance across the post-sampling outcome definitions, with AUC values increasing from 0.619 for 7-day lameness to 0.687 for persistent lameness at both 7 and 28 days. This pattern is biologically plausible because persistent lameness is likely to represent a more stable or clinically meaningful phenotype than a single short-term classification. Importantly, the findings should not be interpreted as showing that OC diagnoses the anatomical source of lameness. Rather, increased OC may indicate heightened bone formation or remodelling activity associated with adaptive response, microdamage repair, or concurrent musculoskeletal pathology.

A notable feature of OC in this dataset was its high negative predictive value at an operational threshold of approximately 1.24 ng/mL (Table 4), which is close to the 75th percentile of the population OC distribution reported in Table 2 (1.42 ng/mL). At this cutoff, OC had limited positive predictive value (0.154–0.316), reflecting the low prevalence of lameness outcomes in this population; given this base rate, a high NPV is expected and does not by itself indicate strong discriminatory ability. Within this study population and outcome prevalence, horses below the threshold were likely to remain sound over the following 28 days, though this may reflect overall lameness prevalence as much as OC’s individual discriminatory value. Such a profile may be more suited to population-level risk stratification than to definitive diagnosis in an individual horse and would require prospective validation before informing specific clinical decisions. The modest AUC values indicate that OC should not be used in isolation as an indicator of soundness status. Whether combining OC with veterinary examination, training history, or other risk indicators improves its utility was not tested in this study and would require prospective evaluation.

The present findings are broadly consistent with prior scintigraphy-based studies in racehorses. In previous work, OC was higher in lame horses than in clinically sound controls and was associated with scintigraphic abnormality category, whereas CTX and the CTX:OC ratio were not consistently associated with 28-day adverse outcome in clinically sound horses [19,20]. Subsequent pooled scintigraphy and radiomics work extended those findings by showing that OC varied across nuclear scintigraphy categories, with scan-positive horses demonstrating a formation-dominant remodelling profile [16]. The current study supports and extends these observations by showing that the association between OC and musculoskeletal outcome is also detectable in a large field cohort of racehorses sampled under routine training conditions, rather than only in horses selected for advanced imaging.

A key contribution of the present study is therefore translational. The present study did not include imaging confirmation for each horse, but it demonstrated that OC retained short-term prognostic value when applied to a larger operational population. This strengthens the interpretation that OC reflects a clinically relevant biological signal rather than an artefact of referral status or imaging-based case selection. At the same time, the more modest AUC values in the present study compared with imaging-supported cohorts are expected because the outcome definition was broader, field-based, and not restricted to confirmed bone pathology. The absolute OC concentrations reported here are markedly lower than those reported by Turlo et al. and Frisbie et al., who used previously validated equine-specific assay platforms, including radioimmunoassay-based methods [12,21]. Direct comparison of absolute osteocalcin concentrations between studies should therefore be undertaken with caution. Osteocalcin immunoassays are recognised to produce substantially different absolute concentrations because of differences in antibody specificity, epitope recognition, assay calibration, and analytical design. This lack of assay harmonisation has been well documented in human medicine, where identical serum samples analysed across multiple commercial osteocalcin assays yielded differences in reported concentrations of up to 130-fold, leading to the conclusion that absolute values are not interchangeable without assay-specific cross-calibration [22]. Accordingly, the reference interval, decision threshold, and concentration values reported in the present study should be interpreted only within the context of the MyBioSource assay used and should not be directly compared with values generated using other analytical platforms.

The lack of useful predictive performance for CTX is also consistent with the earlier studies [16,19,20]. Across the prior scintigraphy work and the present population-level analysis, CTX did not emerge as a reliable short-term indicator of adverse musculoskeletal outcome [20]. This repeated finding suggests that systemic bone resorption markers may be less sensitive to the localised or formation-dominant processes associated with clinically important bone stress in racehorses. However, the strong geographic and surface-related CTX variation observed in the present cohort indicates that CTX remains biologically informative, particularly for understanding how age, training environment, and surface exposure may influence population-level skeletal turnover.

CTX behaved differently from OC. Although CTX is a marker of bone resorption and might be expected to reflect removal of damaged or older bone matrix [14,23], it showed no discriminatory ability for lameness outcomes, with AUC values close to 0.50 for both 7- and 28-day endpoints. CTX also did not differ significantly between sound and lame horses at sampling. These findings suggest that systemic CTX concentration, at least when measured as a single field sample, may not capture the localised skeletal processes most relevant to short-term lameness in racehorses. Alternatively, CTX may be too strongly influenced by background biological and management factors to provide useful individual-level prediction of short-term clinical outcome.

Despite its lack of predictive value for lameness, CTX provided important information about population-level skeletal biology. The most striking source of CTX variability was geographic jurisdiction. NSW horses had markedly higher CTX concentrations than horses sampled in WA or Victoria, and significant variation was also observed across collection venues. The magnitude of these differences suggests that population-wide reference intervals may be insufficient for interpreting CTX in applied racing settings. Jurisdiction-specific or venue-specific interpretive ranges may be required, particularly if differences reflect local training practices, track composition, climate, horse management, sampling timing, or unmeasured environmental exposures. Because samples were processed centrally, the geographic pattern is unlikely to be explained solely by analytical variation.

Training surface was another important contributor to CTX variability. Horses undertaking morning work on polytrack had consistently lower CTX than those trained on sand, grass, or treadmill surfaces, and horses whose main training surface was grass had higher CTX than those trained primarily on sand or polytrack. These findings support the concept that surface properties influence skeletal loading and remodelling responses in racehorses, a notion that is biologically plausible given the mechanosensitive nature of bone adaptation [24,25]. However, training surface is also likely to be confounded by jurisdiction, venue, trainer preference, workload, and horse-level selection factors, including the possibility that trainers preferentially select softer surfaces such as grass or water for horses recovering from or predisposed to lameness. Therefore, the observed association should be interpreted as evidence that surface is a meaningful contributor to bone resorption activity, rather than proof that any specific surface directly causes higher or lower injury risk.

Age-related effects were present for CTX but not OC. Young horses aged 1–2 years had higher CTX than 3-year-old horses, consistent with higher skeletal turnover during maturation and early athletic preparation; horses reach skeletal maturity at approximately 2 years of age, broadly coinciding with the age range sampled in this cohort [26]. This finding is important when interpreting CTX values in mixed-age racing populations because elevated CTX in younger horses may reflect normal developmental and adaptive processes rather than pathology. Sex did not meaningfully affect CTX, although OC differed across sex categories overall, with colts showing the highest median OC concentration; a similar pattern of higher OC in colts relative to fillies has previously been reported in 2-year-old Thoroughbreds, potentially reflecting sex-related differences in osteoblastic activity during the peripubertal period [27]. The weak positive correlation between CTX and OC indicates that the two markers are largely independent and reflect different aspects of bone metabolism. The CTX:OC ratio was highly skewed and did not distinguish sound from lame horses, limiting its immediate clinical usefulness in this dataset.

Neither CTX nor OC was associated with subsequent racing frequency. This is an important distinction because it suggests that biomarker concentrations at the time of sampling reflected skeletal biological state rather than simply the number of races subsequently undertaken. Racing activity is an imperfect proxy for training continuity and health because decisions to race are influenced by performance, scheduling, trainer preference, owner decisions, and commercial considerations. Nevertheless, the lack of association with racing frequency supports the interpretation that OC and CTX should be considered biological markers rather than direct measures of future participation.

Overall, the study supports a role for OC as a biological marker of interest for further investigation into short-term soundness risk in Thoroughbred racehorses. OC appears most useful as a rule-out marker for short-term lameness rather than as a standalone diagnostic test. OC’s discriminatory performance was strongest for persistent lameness in univariate analysis, though this association attenuated after adjustment for other subgroup variables, and its role as a rule-out indicator would require prospective validation before clinical application. CTX appears less useful for short-term prediction but valuable for understanding variation in bone resorption across age groups, jurisdictions, and training surfaces. The findings also establish reference intervals for both markers in a large Australian racing cohort, while highlighting that geographic heterogeneity may limit the application of a single national threshold. Consistent with previous calls for better validation of equine musculoskeletal biomarkers, further prospective work is needed before OC could be considered for use alongside veterinary assessment in practice; at present, its value lies in identifying a population-level biological signal worth continued investigation.

## 5. Limitations

Several limitations should be considered when interpreting these findings. First, the study was observational; therefore, associations between biomarkers, training variables, and outcomes cannot be interpreted as causal. Elevated OC may reflect underlying skeletal remodelling associated with lameness risk, but the study design cannot determine whether increased OC preceded, contributed to, or resulted from the pathological processes leading to lameness.

Second, outcome classification was based on short-term operational follow-up and trainer-reported status and could not be independently verified for all horses. Rectal temperatures were not recorded at the time of sampling. However, all enrolled horses were actively training and considered fit to race by their trainers, with no clinical signs of systemic illness or pyrexia reported; clinically significant elevations in body temperature were therefore considered unlikely in this population. Follow-up data were available for 64.2% of horses at 7 days and 62.6% at 28 days, leaving a substantial proportion with missing outcome data. Horses lost to follow-up may have differed systematically from those with complete data, creating potential response bias. In addition, the categories of sound, lame, spelling, sold or departed, and unknown reflect practical field outcomes rather than standardised diagnostic endpoints.

Third, lameness was treated as an outcome without confirming the anatomical site, tissue type, severity, or imaging diagnosis. Because OC and CTX are systemic markers, they cannot identify whether lameness originated from bone, joint, tendon, ligament, hoof, muscle, or other causes. This limitation may partly explain the modest ROC performance observed for OC and the lack of discriminatory value for CTX.

Fourth, biomarker concentrations were measured at a single timepoint. Bone turnover is dynamic and may vary with circadian rhythm, recent exercise, feeding, recovery time, medication, stress, and stage of training. Serial sampling may provide more clinically useful information than a single measurement, particularly if individual baseline values and within-horse changes can be established.

Fifth, several training variables had incomplete data, including weeks in work and days since last fast work. Although the cohort was large, missingness reduced the effective sample size for some analyses and limited the ability to fully adjust for workload, training intensity, and recent exercise exposure. The study also did not include all potentially relevant confounders, such as detailed training volume, speed profiles, medication history, shoeing, track condition, nutrition, prior injury history, or diagnostic imaging findings.

Finally, the study was conducted in Australian Thoroughbred racehorses across three jurisdictions. The results may not generalise directly to other countries, racing systems, breeds, training environments, or management practices. The high negative predictive value for OC is also dependent on the prevalence of lameness in the sampled population and should be externally validated before being adopted as a universal clinical threshold.

## 6. Conclusions

In this large cohort of fit-to-race Australian Thoroughbred racehorses, OC was associated with concurrent lameness status and showed modest but significant ability to predict short-term lameness outcomes, which attenuated after adjustment for age, sex, state, and training surface. At an operational threshold of approximately 1.24 ng/mL, OC showed high negative predictive value for persistent lameness over the following 28 days, though this may reflect the overall prevalence of lameness in this population as much as OC’s individual discriminatory value. These findings support OC as population-level biological marker warranting further investigation, rather than as a validated screening or diagnostic tool.

CTX did not predict short-term lameness or subsequent racing activity, but it varied substantially by age, jurisdiction, venue, and training surface, indicating that CTX reflects population-level skeletal resorption activity rather than individual short-term lameness risk. The reference intervals generated in this study provide a foundation for future work, but the marked geographic variation observed suggests that jurisdiction-specific interpretation may be required.

Further studies should validate the OC threshold in independent cohorts, incorporate standardised veterinary and imaging diagnoses, and evaluate whether serial biomarker monitoring improves prediction beyond a single measurement. Future work should also explore whether combining OC with clinical examination, training history, surface exposure, and performance data improves its utility for welfare and injury-prevention purposes in Thoroughbred racing.

## Figures and Tables

**Table 1:** Sample characteristics of fit-to-race Thoroughbred racehorses included in the study (*n* = 1359).

Variable	*n* (Valid)	Result
State		
— WA	1359	493 (36.3%)
— NSW		481 (35.4%)
— VIC		385 (28.3%)
Sex		
— Filly	1356	378 (27.9%)
— Mare		192 (14.2%)
— Colt		159 (11.7%)
— Stallion		19 (1.4%)
— Gelding		606 (44.7%)
— Rig		2 (0.1%)
Age (years)		
Age	1356 ^a^	Median 3 (IQR 2–4; range 1–10)
Training characteristics ^b^		
Ambient temperature (°C)	1359	Median 16.0 (IQR 14–18; range 5–27)
Weeks in work	1032	Median 10.0 (IQR 6–16; range 0–66)
Days since last fast work	828	Median 2.0 (IQR 1–4; range 0–227)
Recent lameness at sampling ^c^		
— Sound/bad mover	877	705 (80.4%)
— Lame		57 (6.5%)
— Historic/treating		81 (9.2%)
— Unknown		7 (0.8%)
— Shinsore		15 (1.7%)
— Kissing spine/back		12 (1.4%)
7-day post-sample status		
— Sound	872	697 (79.9%)
— Lame		123 (14.1%)
— Spelling		28 (3.2%)
— Unknown		19 (2.2%)
— Sold/departed		5 (0.6%)
28-day post-sample status		
— Sound	851	579 (68.0%)
— Lame		128 (15.0%)
— Spelling		77 (9.0%)
— Unknown		35 (4.1%)
— Sold/departed		32 (3.8%)

Percentages for categorical variables calculated on valid cases only. ^a^ One-year-old horses were in preparatory training for early two-year-old races commencing in September, consistent with Australian racing practice in which horses turn two on 1 August. ^b^ Training characteristics: Ambient temperature *n* = 1359; weeks in work *n* = 1032 (missing = 327); days since last fast work *n* = 828 (missing = 531). Extended values confirmed by field workers as consistent with horses returning from spelling. ^c^ Recent lameness classified by Racing Australia veterinary staff into six categories. Binary analyses restricted to sound/bad mover (code 1) vs. lame (code 2) are reported in Section 3.4.4 and Section 3.5; remaining categories were excluded from binary comparisons. IQR = interquartile range.

**Table 2:** Percentile distribution and population reference intervals (2.5–97.5 percentile) for CTX and OC/BGLAP in fit-to-race Thoroughbred racehorses (CTX *n* = 1345; OC *n* = 1344).

Percentile	CTX (ng/mL)		OC/BGLAP (ng/mL)		Clinical Interpretation
	Value	95% CI	Value	95% CI	
2.5 ^a^	0.13	0.10–0.15	0.02	0.01–0.05	Lower reference limit
5	0.19	—	0.06	—	
10	0.37	—	0.14	—	
25	0.56	—	0.39	—	Lower quartile
50	1.15	—	0.75	—	Median
75	3.95	—	1.42	—	Upper quartile
90	6.01	—	3.87	—	
95	7.95	—	5.26	—	
97.5 ^a^	9.44	8.10–10.18	7.25	5.68–10.66	Upper reference limit

CTX = C-terminal telopeptide of type I collagen; OC = osteocalcin/BGLAP; CI = confidence interval. ^a^ 95% CI around reference limits calculated using the nonparametric rank-based method (Reed et al.): rank = *n* × *p* ± 1.645 × √(*n* × *p* × (1 − *p*)); CTX *n* = 1345, OC *n* = 1344. Percentiles calculated using the weighted average method (HAVERAGE) in SPSS v26, consistent with ASVCP reference interval guidelines. — = 95% CI not calculated for interior percentiles.

**Table 3:** Comparisons of CTX and OC/BGLAP concentrations across subgroups in fit-to-race Thoroughbred racehorses (*n* = 1359).

Subgroup	*n*	CTX Result	OC Result	*p*-Value
Age group ^a^				
Young (1–2 years)	343–345	Median 1.36 (95% CI 1.20–2.08); IQR 0.70–4.09	Median 0.84 (95% CI 0.75–0.92); IQR 0.41–1.37	
Mid (3 years)	436–439	Median 0.97 (95% CI 0.82–1.17); IQR 0.54–3.71	Median 0.71 (95% CI 0.66–0.80); IQR 0.36–1.48	
Mature (4 years)	265	Median 0.99 (95% CI 0.85–1.20); IQR 0.57–3.91	Median 0.77 (95% CI 0.68–0.88); IQR 0.38–1.35	
Senior (5+ years)	295	Median 1.20 (95% CI 0.87–1.42); IQR 0.53–4.10	Median 0.73 (95% CI 0.64–0.81); IQR 0.42–1.40	
Kruskal–Wallis		H = 9.751, df = 3	H = 1.764, df = 3	CTX: 0.021 * OC: 0.623
Post hoc ^b^: Young vs. Mid		U = 65,767, Z = −3.038	—	CTX: 0.002 *
Sex				
Filly	371–373	Median 1.17 (95% CI 0.97–1.20); IQR 0.55–3.60	Median 0.69 (95% CI 0.63–0.75); IQR 0.35–1.24	
Mare	191–192	Median 1.20 (95% CI 0.92–2.14); IQR 0.57–4.37	Median 0.75 (95% CI 0.63–0.90); IQR 0.37–1.34	
Colt	158	Median 1.78 (95% CI 1.29–2.92); IQR 0.71–4.14	Median 0.93 (95% CI 0.76–1.07); IQR 0.49–1.56	
Stallion	19	Median 1.13 (95% CI 0.55–3.36); IQR 0.41–3.44	Median 0.66 (95% CI 0.32–1.03); IQR 0.31–1.05	
Gelding	598–600	Median 1.05 (95% CI 0.92–1.20); IQR 0.56–3.96	Median 0.78 (95% CI 0.70–0.85); IQR 0.41–1.55	
Rig	2	Median 0.63 (descriptive only, *n* = 2)	Median 1.22 (descriptive only, *n* = 2)	
Kruskal–Wallis		H = 7.478, df = 5	H = 11.991, df = 5	CTX: 0.187 OC: 0.035 *
Post hoc ^b^: Filly vs. Colt		—	U = 24,171, Z = −3.193	OC: 0.001 *
State ^c^				
WA	484–486	Median 0.65 (95% CI 0.61–0.70); IQR 0.46–2.27	Median 0.73 (95% CI 0.66–0.81); IQR 0.31–1.43	
NSW	471–476	Median 3.21 (95% CI 2.78–3.67); IQR 1.10–4.94	Median 0.81 (95% CI 0.75–0.90); IQR 0.49–1.38	
VIC	382–385	Median 1.07 (95% CI 0.99–1.15); IQR 0.66–2.89	Median 0.70 (95% CI 0.64–0.82); IQR 0.36–1.44	
Kruskal–Wallis		H = 155.656, df = 2	H = 6.816, df = 2	CTX: <0.001 *** OC: 0.033 *
Post hoc ^b^: WA vs. NSW		Z = −11.626	Z = −2.354	CTX: <0.001 OC: 0.019 ns
Post hoc ^b^: WA vs. VIC		Z = −5.571	Z = −0.540	CTX: <0.001 OC: 0.589 ns
Post hoc ^b^: NSW vs. VIC		Z = −7.963	Z = −2.054	CTX: <0.001 OC: 0.040 ns
Racing region ^d^				
Kruskal–Wallis (12 regions)		H = 524.976, df = 11	H = 131.422, df = 11	Both <0.001 ***
Recent lameness at time of sampling ^e^				
Sound/bad mover	694–698	Median 0.94 (95% CI 0.88–1.07); IQR 0.53–3.23 (CTX vs. Lame)	Median 0.67 (95% CI 0.62–0.73); IQR 0.31–1.23 (OC vs. Lame)	
Lame	55–57	Median 1.20 (95% CI 0.83–3.56); IQR 0.60–4.69 (CTX vs. Sound)	Median 1.26 (95% CI 0.78–1.66); IQR 0.64–2.17(OC vs. Sound)	
Historic/treating	79–81	Median 0.67 (95% CI 0.54–0.81); IQR 0.42–1.28	Median 0.49 (95% CI 0.35–0.74); IQR 0.16–1.02	
Unknown	7	Median 0.45 (95% CI 0.34–7.68); IQR 0.34–1.20	Median 1.40 (95% CI 0.84–3.01); IQR 0.84–3.01	
Shinsore	14–15	Median 0.47 (95% CI 0.34–0.70); IQR 0.30–0.70	Median 1.34 (95% CI 0.69–1.90); IQR 0.68–2.50	
Kissing spine/back	12	Median 0.60 (95% CI 0.44–0.85); IQR 0.44–0.85	Median 0.65 (95% CI 0.29–1.80); IQR 0.25–1.67	
Mann–Whitney: Sound vs. Lame		U = 16,164.5, Z = −1.891	U = 13,589.0, Z = −3.982	CTX: 0.059 OC: <0.001 ***
Main training surface				
Sand	701–702	Median 0.87 (95% CI 0.78–0.96); IQR 0.50–3.34	Median 0.70 (95% CI 0.66–0.77); IQR 0.34–1.39	
Poly	192–193	Median 0.86 (95% CI 0.74–1.01); IQR 0.44–1.54	Median 0.75 (95% CI 0.64–0.89); IQR 0.36–1.31	
Grass	53–56	Median 1.95 (95% CI 1.20–3.36); IQR 1.20–4.96	Median 0.74 (95% CI 0.52–1.12); IQR 0.35–1.62	
Kruskal–Wallis		H = 30.893, df = 2	H = 1.481, df = 2	CTX: <0.001 *** OC: 0.477
Post hoc ^b^: Sand vs. Poly		U = 61,612, Z = −1.928	—	CTX: 0.054 ns
Post hoc ^b^: Sand vs. Grass		U = 11,072.5, Z = −4.919	—	CTX: <0.001 ***
Post hoc ^b^: Poly vs. Grass		U = 2553, Z = −5.588	—	CTX: <0.001 ***
Morning work type ^f^				
Off	113–115	Median 2.10 (95% CI 1.20–3.35); IQR 1.20–4.88	Median 0.63 (95% CI 0.47–0.80); IQR 0.31–1.57	
Walk	36–38	Median 0.81 (95% CI 0.50–1.20); IQR 0.44–1.70	Median 0.79 (95% CI 0.64–1.24); IQR 0.39–1.42	
Swim	49	Median 0.69 (95% CI 0.56–1.07); IQR 0.44–1.57	Median 0.68 (95% CI 0.55–0.96); IQR 0.37–1.44	
Treadmill	49–50	Median 1.18 (95% CI 0.69–2.22); IQR 0.52–3.49	Median 0.71 (95% CI 0.48–1.02); IQR 0.37–1.58	
Slow	305–309	Median 0.76 (95% CI 0.67–0.89); IQR 0.50–2.80	Median 0.72 (95% CI 0.64–0.83); IQR 0.30–1.37	
Fast	433–435	Median 0.86 (95% CI 0.80–0.94); IQR 0.46–1.65	Median 0.67 (95% CI 0.61–0.78); IQR 0.34–1.25	
Mixed	32	Median 1.47 (95% CI 0.77–3.76); IQR 0.63–4.70	Median 0.63 (95% CI 0.33–1.06); IQR 0.25–1.52	
Kruskal–Wallis		H = 67.026, df = 6	H = 14.272, df = 6	CTX: <0.001 *** OC: 0.027 *
Morning training surface ^f^				
Sand	530–535	Median 0.87 (95% CI 0.77–0.96); IQR 0.49–3.05	Median 0.71 (95% CI 0.65–0.80); IQR 0.34–1.37	
Poly	189–191	Median 0.72 (95% CI 0.62–0.81); IQR 0.43–1.06	Median 0.69 (95% CI 0.61–0.83); IQR 0.35–1.15	
Grass	63–64	Median 1.15 (95% CI 0.94–1.85); IQR 0.72–4.32	Median 0.78 (95% CI 0.41–0.97); IQR 0.27–1.82	
Treadmill	49–50	Median 1.14 (95% CI 0.69–1.64); IQR 0.52–3.35	Median 0.71 (95% CI 0.49–1.02); IQR 0.38–1.60	
Water	47–48	Median 0.69 (95% CI 0.56–1.06); IQR 0.44–1.36	Median 0.66 (95% CI 0.53–0.96); IQR 0.35–1.44	
Mixed	20	Median 0.93 (95% CI 0.63–1.95); IQR 0.58–2.00	Median 0.38 (95% CI 0.18–0.73); IQR 0.18–0.78	
Kruskal–Wallis		H = 32.162, df = 5	H = 4.689, df = 5	CTX: <0.001 *** OC: 0.455
Post hoc ^g^: Sand vs. Poly		U = 40,103.5, Z = −4.259	—	CTX: <0.001 ***
Post hoc ^g^: Poly vs. Grass		U = 3554.0, Z = −5.009	—	CTX: <0.001 ***
Post hoc ^g^: Poly vs. Treadmill		U = 3407.0, Z = −3.117	—	CTX: 0.002 *
Post hoc ^g^: Grass vs. Water		U = 1008.0, Z = −3.104	—	CTX: 0.002 *
CTX:OC ratio by state (exploratory) ^h^				
WA	477	Median ratio: 1.36	—	
NSW	471	Median ratio: 3.26	—	
VIC	382	Median ratio: 1.48	—	
Kruskal–Wallis		H = 45.496, df = 2	—	<0.001 ***
Spearman correlations with continuous variables				
Weeks in work	1019–1020	rs = 0.080	rs = −0.054	CTX: 0.010 * OC: 0.087
Days since last fast work	819–821	rs = 0.064	rs = 0.022	CTX: 0.066 OC: 0.526
Ambient temperature (°C)	1344–1345	rs = 0.030	rs = 0.081	CTX: 0.269 OC: 0.003 *

CTX = C-terminal telopeptide of type I collagen; OC = osteocalcin/BGLAP; ns = not significant after Bonferroni correction. ^a^ Kruskal–Wallis test across four age groups. Only significant post hoc comparison shown. ^b^ Post hoc Mann–Whitney U tests with Bonferroni correction. Thresholds: Age group *p* < 0.0083 (6 pairs); Sex *p* < 0.005 (10 pairs); State *p* < 0.0167 (3 pairs); Main surface *p* < 0.0167 (3 pairs). ^c^ OC state differences did not survive Bonferroni correction (all OC post hoc *p* > 0.017). ^d^ Region-level Kruskal–Wallis across 12 regions. Albany (WA, *n* = 1) excluded from regional interpretation. Post hoc pairwise comparisons not performed (66 pairs). ^e^ Recent lameness binary Mann–Whitney restricted to sound/bad mover (code 1) vs. lame (code 2). The *n* values shown reflect the number of horses with valid BTM data within each code. Remaining categories reported descriptively and excluded from binary comparison. 95% confidence intervals for the unknown and kissing spine/back categories are wide, reflecting their small sample sizes (*n* = 7 and *n* = 12, respectively). ^f^ Morning work type: post hoc not performed (21 pairs, threshold *p* < 0.0024). Morning training surface OC: non-significant overall (*p* = 0.455), post hoc not performed. ^g^ Morning training surface CTX post hoc: Bonferroni threshold *p* < 0.0033 (15 pairs). Four significant pairs are shown. ^h^ CTX:OC ratio = CTX/OC; *n* = 1330. Exploratory analysis. Median reported as central tendency measure given extreme skewness (skewness = 21.2). Significance codes: * *p* < 0.05; *** *p* < 0.001.

**Table 4:** Post-sample outcome frequencies and associations between CTX and OC/BGLAP concentrations and subsequent soundness and racing activity in fit-to-race Thoroughbred racehorses.

Outcome	*n* Sound	*n* Lame	CTX (ng/mL)	OC (ng/mL)	Statistics
Post-sample outcome frequencies					
7-day follow-up	697 (79.9%)	123 (14.1%)	Unknown: 19 (2.2%) Spelling: 28 (3.2%) Sold/Departed: 5 (0.6%)		
28-day follow-up	579 (68.0%)	128 (15.0%)	Unknown: 35 (4.1%) Spelling: 77 (9.0%) Sold/Departed: 32 (3.8%)		
Biomarker concentrations by 7-day post-sample status (Sound vs. Lame) ^a^					
Sound	692	—	Median 0.89 (95% CI 0.81–0.96); IQR 0.50–3.01	Median 0.62 (95% CI 0.57–0.67); IQR 0.29–1.12	
Lame	—	123	Median 0.82 (95% CI 0.72–1.20); IQR 0.49–3.87	Median 0.99 (95% CI 0.72–1.38); IQR 0.44–2.23	
Mann–Whitney U			U = 41,590.5, Z = −0.251	U = 30,956.5, Z = −4.578	CTX: *p* = 0.802 OC: *p* < 0.001 ***
Biomarker concentrations by 28-day post-sample status (Sound vs. Lame) ^a^					
Sound	575	—	Median 0.84 (95% CI 0.75–0.91); IQR 0.49–3.24	Median 0.66 (95% CI 0.62–0.72); IQR 0.31–1.13	
Lame	—	126	Median 0.84 (95% CI 0.71–1.20); IQR 0.47–2.86	Median 0.84 (95% CI 0.70–1.24); IQR 0.36–1.83	
Mann–Whitney U			U = 35,390.5, Z = −0.224	U = 29,785.5, Z = −3.128	CTX: *p* = 0.823 OC: *p* = 0.002 **
ROC curve analysis—discriminatory ability of OC/BGLAP for lameness outcomes ^b^					
7-day outcome (CTX)	—	—	AUC = 0.499 (0.444–0.553)	—	*p* = 0.961
7-day outcome (OC)	—	—	—	AUC = 0.619 (0.562–0.675)	*p* < 0.001 ***
28-day outcome (CTX)	—	—	AUC = 0.489 (0.434–0.545)	—	*p* = 0.703
28-day outcome (OC)	—	—	—	AUC = 0.568 (0.512–0.625)	*p* = 0.014 *
Diagnostic performance of OC/BGLAP at optimal cutoff (~1.24 ng/mL, Youden index) ^c^					
Analysis	Se (95% CI)		Sp (95% CI)	PPV (95% CI)	NPV (95% CI)
12a: 7-day Lame only (*n* = 820)	0.471 (0.384–0.560)		0.769 (0.736–0.799)	0.263 (0.209–0.325)	**0.893 (0.865–0.915)**
12b: 7-day Lame + Spelling (*n* = 848)	0.480 (0.401–0.560)		0.777 (0.745–0.807)	0.316 (0.258–0.379)	**0.875 (0.846–0.899)**
12c: 28-day Lame + Spelling (*n* = 784)	0.421 (0.338–0.508)		0.744 (0.707–0.778)	0.265 (0.209–0.330)	**0.854 (0.821–0.882)**
12d: Persistent lameness (*n* = 749)	0.529 (0.395–0.659)		0.786 (0.754–0.815)	0.154 (0.108–0.215)	**0.958 (0.938–0.971)**
Correlations between biomarker concentrations and subsequent racing activity ^d^					
Races between 28–56 days	836–839	—	rs = 0.061, *p* = 0.076	rs = 0.039, *p* = 0.254	Both ns
Races within 60 days	836–839	—	rs = 0.060, *p* = 0.085	rs = 0.016, *p* = 0.652	Both ns

CTX = C-terminal telopeptide of type I collagen; OC = osteocalcin/BGLAP; AUC = area under the curve; Se = sensitivity; Sp = specificity; PPV = positive predictive value; NPV = negative predictive value; ns = not significant; CI = 95% Wilson score confidence interval. ^a^ Mann–Whitney U test comparing sound vs. lame horses only. Horses classified as unknown, spelling, or sold/departed excluded. Outcome data self-reported by trainers and could not be independently verified. ^b^ ROC curve analysis; AUC values reported with asymptotic 95% CI under the nonparametric assumption. Positive state = lame (code 2). Larger OC values indicate stronger evidence for a positive state. ^c^ Optimal cutoff identified by Youden index (Se + Sp − 1) from ROC coordinate tables. Cutoffs: 12a = 1.2404; 12b = 1.2150; 12c = 1.2357; 12d = 1.2542 ng/mL. 2 × 2 contingency tables: 12a TP = 57, FN = 64, TN = 532, FP = 160; 12b TP = 71, FN = 77, TN = 538, FP = 154; 12c TP = 53, FN = 73, TN = 428, FP = 147; 12d TP = 27, FN = 24, TN = 544, FP = 148. Persistent lameness (12d) defined as lame at both 7-day AND 28-day follow-up. ROC positive *n*: 12a = 121, 12b = 148, 12c = 126, 12d = 51 (reflects horses with valid OC within each outcome group). NPV values in bold represent the primary clinical metric of interest. ^d^ Spearman rank correlations. *n* reflects the number of horses with available racing activity data. Follow-up data available for *n* = 872 horses at 7 days (64.2%) and *n* = 851 horses at 28 days (62.6%). Missing follow-up reflects non-response by trainers. Significance codes: * *p* < 0.05; ** *p* < 0.01; *** *p* < 0.001.

## Data Availability

The data that support the findings of this study are openly available at https://www.dropbox.com/scl/fi/u5r5npto10q5t4ff7202m/ALL-BLOODS-RA-Data_Updated.xlsx?rlkey=x4iugy3j7jl3409jntezb578w&st=gkj0lo6c&dl=0 accessed on 11 August 2026.
